# Rapid reduction of maternal mortality in Uganda and Zambia through the saving mothers, giving life initiative: results of year 1 evaluation

**DOI:** 10.1186/s12884-017-1222-y

**Published:** 2017-01-19

**Authors:** Florina Serbanescu, Howard I. Goldberg, Isabella Danel, Tadesse Wuhib, Lawrence Marum, Walter Obiero, James McAuley, Jane Aceng, Ewlyn Chomba, Paul W. Stupp, Claudia Morrissey Conlon

**Affiliations:** 1 Division of Reproductive Health, Centers for Disease Control and Prevention, 4770 Buford Hwy, Atlanta, GA 30341 USA; 2 Uganda Country Office, Centers for Disease Control and Prevention, Plot 51-59 Nakiwogo Road, Entebbe, Uganda; 3 Ministry of Health, Zambia Country Office, Centers for Disease Control and Prevention, 351 Independence Avenue, Lusaka, Zambia 10101; 4 Uganda Ministry of Health, 6 Lourdel Road, Wandegeya, Kampala Uganda; 5 Zambia Ministry of Health, Ndeke House, Haile Selassie Avenue, Lusaka, Zambia; 6 United States Agency for International Development, 2100 Crystal Drive, Arlington, VA 22202 USA; 7 Saving Mothers Giving Life Research Group, Atlanta, USA; 8 Field Support Branch, Division of Reproductive Health, National Center for Chronic Disease Prevention and Health Promotion, Centers for Disease Control and Prevention, 4770 Buford Hwy, NE, MS F74, Atlanta, GA 30341 USA

**Keywords:** Maternal mortality, Pregnancy complications, Emergency obstetric care, Verbal autopsy, Low-resource countries, Sub-Saharan Africa

## Abstract

**Background:**

Achieving maternal mortality reduction as a development goal remains a major challenge in most low-resource countries. Saving Mothers, Giving Life (SMGL) is a multi-partner initiative designed to reduce maternal mortality rapidly in high mortality settings through community and facility evidence-based interventions and district-wide health systems strengthening that could reduce delays to appropriate obstetric care.

**Methods:**

An evaluation employing multiple studies and data collection methods was used to compare baseline maternal outcomes to those during Year 1 in SMGL pilot districts in Uganda and Zambia. Studies include health facility assessments, pregnancy outcome monitoring, enhanced maternal mortality detection in facilities, and population-based investigation of community maternal deaths. Population-based evaluation used standard approaches and comparable indicators to measure outcome and impact, and to allow comparison of the SMGL implementation in unique country contexts.

**Results:**

The evaluation found a 30% reduction in the population-based maternal mortality ratio (MMR) in Uganda during Year 1, from 452 to 316 per 100,000 live births. The MMR in health facilities declined by 35% in each country (from 534 to 345 in Uganda and from 310 to 202 in Zambia). The institutional delivery rate increased by 62% in Uganda and 35% in Zambia. The number of facilities providing emergency obstetric and newborn care (EmONC) rose from 10 to 25 in Uganda and from 7 to 11 in Zambia. Partial EmONC care became available in many more low and mid-level facilities. Cesarean section rates for all births increased by 23% in Uganda and 15% in Zambia. The proportion of women with childbirth complications delivered in EmONC facilities rose by 25% in Uganda and 23% in Zambia. Facility case fatality rates fell from 2.6 to 2.0% in Uganda and 3.1 to 2.0% in Zambia.

**Conclusions:**

Maternal mortality ratios fell significantly in one year in Uganda and Zambia following the introduction of the SMGL model. This model employed a comprehensive district system strengthening approach. The lessons learned from SMGL can inform policymakers and program managers in other low and middle income settings where similar approaches could be utilized to rapidly reduce preventable maternal deaths.

## Background

Maternal mortality remains high in sub-Saharan Africa (SSA) despite recent efforts to accelerate reduction [1–3]. The maternal mortality ratio (MMR) in SSA is estimated to have declined by 49% since 1990, however, at 510 maternal deaths per 100,000 live births, remains the highest regional maternal mortality of the world. It is almost three times higher than in Southern Asia and 30 times higher than in high-resource regions [4, 5]. The great majority of maternal deaths are preventable through effective, low-cost interventions [6–11].

Saving Mothers, Giving Life (SMGL) is a multi-partner initiative designed to rapidly reduce deaths stemming from pregnancy and childbirth through a comprehensive set of evidence-based interventions in high-mortality, low-resource settings. It established an ambitious target of a 50% decline in the MMR in one year to address the need for accelerated progress to meet the fifth Millennium Development Goal (MDG5) of a 75% reduction in MMR by 2015.

SMGL draws upon the investment and expertise of public and private organizations and existing infrastructure, partnerships, and services, including US Government platforms for combating HIV/AIDS and improving maternal and child health [12, 13]. SMGL is based on a health district-strengthening model that is planned and carried out in close collaboration with the national Governments of Uganda and Zambia and with local governments in SMGL districts. Other current partners are the US Government [Centers for Disease Control and Prevention (CDC), US Agency for International Development (USAID), US Peace Corps, US Department of Defense, and US Office of the Global AIDS Coordinator], the American College of Obstetricians and Gynecologists, Every Mother Counts, Merck for Mothers, the Government of Norway, and Project CURE.

The SMGL model aims for rapidly achievable, measurable, sustainable reductions in maternal and newborn mortality and Prevention of Maternal to Child Transmission of HIV. It employs a comprehensive approach that strengthens district health systems to ensure that every pregnant woman has access to safe basic delivery services and, in the event of an obstetric complication, life-saving emergency obstetric and newborn care (EmONC) [14] within 2 h. The model builds upon existing district health strategies and platforms to address the “Three Delays”, i.e., delays in: seeking appropriate services; reaching those services; and receiving timely, quality care at the facility [15]. It promotes and closely monitors evidence-based interventions in facilities around the time of labor, delivery and immediately postpartum, [16–20] when earlier studies estimate that most maternal deaths and about half of newborn deaths occur [21–26]. In collaboration with the governments of Uganda and Zambia, SMGL has introduced in 2012 a wide range of interventions in communities and health facilities (public and private) in 4 pilot districts in each country. These included: a) demand generation for antenatal, facility delivery, postpartum care and raising awareness about birth planning, pregnancy complications, HIV testing and treatment and postpartum family planning services; b) facility upgrading and equipping, provisions of medical commodities and supplies including safe blood, and hiring, training and mentoring mid and high level staffing to increase the number and geographical distribution of quality basic and comprehensive EmONC services with 24 h coverage; c) strengthening linkages between communities and facilities through integrated communications and transportation systems and opening of new maternity waiting homes; and d) increasing capacity of district health office personnel and facility personnel for data collection, management and use and thus strengthening host country health management information systems (Table 1).

**Table 1:** SMGL Interventions Implemented in Uganda and Zambia to reduce the Three Delays

Increase Awareness and Seeking Care for Safe Delivery (to reduce the First Delay)
• Training of Village Health Teams to encourage birth preparedness and increase demand for facility-based delivery care
• Community outreach activities to counsel women, families, local leaders, and community organizations on the importance of birth planning, recognition of danger signs of pregnancy complications, attending at least 4 antenatal care visits, facility delivery care, HIV testing and treatment, post-partum homecare for mother/newborn and postpartum family planning.
• Distribution of Mama Kits to incentivize facility-based births • Community mobilization messages (radio, billboards, newspaper articles) and drama skits
• Promotion of demand- and supply-side financial incentives to facilitate women seeking, accessing and utilizing quality care services (eg. transport and delivery care vouchers, user-fee reductions, and conditional cash transfers).
Increase access to quality health care services (to reduce the Second Delay)
• Upgrade a sufficient number of public and private facilities with appropriate geographical positioning to provide—24 h per day/7 days a week—clean and safe basic delivery services, quality HIV testing, counseling and treatment (for woman, partner, and baby as appropriate), and essential newborn care for all pregnant women in the district.
• Ensure that a minimum of five emergency obstetric and newborn care (EmONC) facilities (public and private), including at least one facility that can provide comprehensive EmONC per 500,000 population are providing the recommended life-saving obstetric interventions 24 h per day/7 days a week.
• Hire a sufficient number^a^ of skilled birth attendants to provide, on a consistent basis, quality respectful basic delivery care, diagnosis and stabilization of complications, and if needed, timely facilitated referral for EmONC. Performance-based EmONC-trained personnel in facilities that provide basic and comprehensive EmONC.
• Create a 24-h/7 day per week, consultative, protocol-driven, quality-assured, integrated (public and private) communication/transportation referral system that ensures women with complications reach emergency services within 2 h. This includes providing, where appropriate, temporary lodging in maternity waiting homes for women with high-risk pregnancies or who live greater than 2-h travel time to an EmONC facility.
Improve quality, appropriate and respectful care (to reduce the Third Delay)
• Train health professionals in emergency obstetric care, including obstetric surgeries
• Ensure mentoring of newly hired personnel and supported supervision
• Strengthen supply chains for essential supplies and medicines
• Ensure implementation of quality, effective interventions to prevent and treat obstetric complications (MgSO4, infection prevention practices, assisted vaginal delivery, Active Management of the Third Stage of Labor [AMSTL], C-section and other obstetric surgeries (e.g., laparotomy, hysterectomy, repairs following obstetric complications), safe blood supplies, prevention of HIV maternal to child transmission, etc.)
• Introduce sound managerial practices utilizing ‘short-loop’ data feedback and response, to ensure reliable delivery of quality essential and emergency maternal and newborn care.
• Strengthen maternal mortality surveillance in communities and facilities, including timely, no-fault, medical death reviews performed in follow-up to every institutional maternal death with cause of death information used for ongoing monitoring and quality improvement.
• Promote a government-owned HMIS data-gathering system that accurately records every birth, obstetric and newborn complication and treatment provided, and birth outcomes at public and private facilities in the district. Where appropriate, m-health approaches to facilitate the monitoring activities.

^a^WHO guidelines recommend 1 midwife per 120 deliveries/year; 1–2 doctors and 6 medical personnel (midwives, clinical officers, and nurses) for every 1000 births.

The SMGL districts in Uganda and Zambia differ in many important ways (Table 2) [27, 28]. Ugandan districts have more hospitals and health centers with surgical capacity, including a regional hospital that covers 7 districts. In both countries, most hospitals are designated to perform comprehensive obstetric care, including surgeries. Health centers are generally designed to provide basic emergency obstetric care. Health posts provide routine delivery care and refer complicated births to higher level facilities. District-wide facility and community SMGL activities were geared toward increasing access to and availability of quality obstetric care (Table 3) [29, 30].

**Table 2:** Selected national and SMGL Districts Indicators before Interventions

Characteristic	Uganda	Zambia
National Indicators		
Life expectancy at birth (male/female) (2012)^a^	56/58	55/58
Health Expenditures		
Total expenditure on health as % of GDP (2011)^a^	9.3	6.2
Total expenditure on health as % of general government expenditures^a^	10.1	16.4
SMGL 4-District Indicators		
Area (sq. km)	10,851	49,468
Population (2011)^b^	1,750,000	925,198
% of Population in rural areas	84%	61%
Women of Reproductive Age^b^	342,060	193,515
Expected Live Births^c^	78,261	37,267
Type of Health Care Facility^d^		
Health Posts	19	16
Health centers without surgical care	72	91
Health centers with surgical care	8	0
District Hospitals	7	6
Regional Hospital^e^	1	0
Facility Ownership^f^		
Government	65	106
Private for profit	11	0
Private not for profit	31	7
Emergency Obstetric and Newborn Care (EmONC) Facilities^f^		
Comprehensive EmONC	7	4
Basic EmONC	3	3

^a^ *GDP* Gross Domestic Product. Source: World Health Statistics, 2014

^b^Based on the district-wide census of the population conducted in 2013 in Uganda (4 districts) and in 2012 in Zambia (4 districts) and projected back to 2011 [31]

^c^Estimated by summing the expected births in each age group (number of women of reproductive age from district-wide census multiplied by their age specific fertility rates from 2011 DHS) in Uganda and by applying 2010 Census crude birth rates in Zambia

^d^Health facilities providing delivery care prior to SMGL [29]

^e^Fort Portal is the regional referral hospital located in Kabarole district; it has 351 beds and serves the entire Ruwenzori region constituted of 3 SMGL-supported districts (Kabarole, Kyenjojo, Kamwenge) and 4 non-SMGL districts (Kasese, Ntoroko, Kyegegwa and Bundibujyo)

^f^EmONC includes a set of life-saving interventions (aka “signal functions”) that the World Health Organization has recommended to reduce maternal and neonatal mortality (WHO, 2009). Basic EmONC interventions include administration of parenteral antibiotics, uterotonics, or anticonvulsants; manual removal of placenta; removal of retained products; assisted vaginal delivery; and basic neonatal resuscitation. Comprehensive care interventions include two additional services: ability to perform obstetric surgery (e.g., C- section) and blood transfusion. Facilities were classified based on whether they had, within the previous 3 months, performed each of these interventions. Because assisted vaginal delivery—using either forceps or vacuum extractor—is relatively uncommon in both Uganda and Zambia, some facilities were classified as fully providing EmONC care even if they did not perform assisted vaginal deliveries within the past 3 months (EmONC-1)

Note 1: in Uganda, district and regional hospitals and health centers with surgical capacity (health centers IV) are designated as CEmONC facilities, able to perform each of the 9 signal functions and serving about 100,000 population [27]; in Zambia, only district and higher level hospitals are designated to provide CEmONC care [28]

Note 2: Unless otherwise noted, the figures in the table are numbers

**Table 3:** Types of facility and community interventions, accomplishments, and resources added during SMGL Year 1

	Gains during Year 1	
	Uganda	Zambia
Infrastructure developed		
operating theaters built or renovated	8	0
facilities with electricity upgrades	35	22
facilities with uninterrupted water supply added	6	10
mother shelters built or renovated	4	11
Human Resources added		
medical officers	18	0
obstetricians	0	0
clinical officers	15	0
nurses	20	0
midwives	103	19
Health providers who received EmONC training	316	199
Supply-chain system improvements		
facilities that received EmONC equipment	111	122
facilities that received essential commodities and supplies	89	122
facilities with protocols for clinical mgmt. of obstetric complications complications	57	NA
Communication-Transportation Added		
vehicle ambulances	7	5
motorcycle ambulances (E-rangers)	16	14
bicycles	1	46
Vouchers redeemed for institutional delivery^a^		
transportation vouchers	29,436	NA
private care vouchers (also cover transportation)	85	NA
Community-based efforts added		
community volunteers^b^	4076	1548
community mobilization events	701	6
radio spots broadcast	36,146	3807

^a^Transportation vouchers introduced in 3 districts and private care vouchers in all 4 districts in Uganda; vouchers were not introduced in Zambia

^b^Includes village health teams (VHTs)—one per community in Uganda, trained to provide preventive MCH services and conduct surveillance activities—and Safe Motherhood Action Groups (SMAGs) in Zambia, recruited and trained to link communities with facility-based care

Note: All figures in the table are numbers

This paper provides key results for Year 1 of the SMGL initiative that serve as “proof of concept” and as the basis for scale-up in Uganda, Zambia and potentially additional countries. The focus is on maternal mortality reduction and its principal determinants. Details of process-related results are reported elsewhere [29].

## Methods

SMGL used multiple monitoring and evaluation processes with varied data sources (Table 4). Comparisons of maternal and perinatal outcomes were made between a baseline period (June 2011–May 2012) and Year 1 (June 2012–May 2013) after full implementation of SMGL interventions. Key indicators compared include: EmONC process and outcome monitoring indicators, [14] maternal mortality ratios in facilities in both countries, as well as population-based maternal mortality measurements in Uganda [31].

**Table 4:** SMGL data sources by groups of indicators

Period and Indicator	Uganda			Zambia	
	Community	Health Center IV and Hospitals	Health Centers III and II	Community	Health Centers and Hospitals
Baseline (June 2011–May 2012)					
Routine and Emergency Obstetric Care Indicators	NA	Facility Assessment	Facility Assessment	NA	Facility Assessment
Institutional Deliveries	NA	Individual Outcome Data (POM)	Enhanced Aggregate Outcome Data	NA	Enhanced Aggregate Outcome Data
AMTSL use	NA	Individual Outcome Data (POM)	Enhanced Aggregate Outcome Data	NA	NA
Direct Obstetric Complications Prevalence Rates	NA	Individual Outcome Data; Triangulation of facility registers	Enhanced Aggregate Outcome Data	NA	Enhanced Aggregate Outcome Data
Case Specific Maternal Mortality and Case Fatality Rates	RAMOS	RAPID	Enhanced Aggregate Outcome Data	4-distirct Census^a^	Individual Maternal Deaths
Population Maternal Mortality	RAMOS	NA	NA	4-distirct Census^a^	NA
Year 1 (June 2012–May 2013)					
Routine and Emergency Obstetric Care Indicators	NA	Facility Assessment	Facility Assessment	NA	Facility Assessment
Institutional Deliveries	NA	Individual Outcome Data (POM)	Enhanced Aggregate Outcome Data	NA	Enhanced Aggregate Outcome Data
AMTSL use	NA	Individual Outcome Data (POM)	Enhanced Aggregate Outcome Data	NA	NA
Direct Obstetric Complications Prevalence Rates	NA	Individual Outcome Data; Triangulation of facility registers	Enhanced Aggregate Outcome Data	NA	Enhanced Aggregate Outcome Data
Case Specific Maternal Mortality and Case Fatality Rates	RAMOS	RAPID	Enhanced Aggregate Outcome Data	SMAG Reporting^b^	Individual Maternal Deaths
Population Maternal Mortality	RAMOS	NA	NA	NA	NA

^a^Conducted in 2012; population maternal mortality rates were estimated at baseline but comparable data collection at the end of Year 1 was not conducted

^b^Safe Motherhood Action Groups started to report community maternal deaths in 2013 but they cover less than a third of population of the 4 districts in Zambia

Overall and cause-specific maternal mortality were calculated after classifying identified deaths using the World Health Organization (WHO)’s maternal mortality application of the ICD-10 (ICD-MM) [32]. We calculated MMRs in facilities in both countries (using the number of live births in facilities as the denominator) and population-based MMR in Uganda (using the estimated number of live births in the SMGL districts).

A brief overview of the data sources is presented below:

### Health facility assessments (HFA)

Each country conducted a baseline and Year 1 HFA of every facility that provided delivery care in the SMGL districts, whether public, private, or faith-based, using a modified version of the standard point-in-time EmONC questionnaire [33]. Baseline data were collected by trained personnel several months prior to SMGL implementation (January 2012 in Zambia and March 2012 in Uganda). These data were used to assess current facility capacity to perform life-saving EmONC interventions [29]. The assessment of all delivering facilities in all districts allowed for a rational distribution of human and financial resources to strengthen the safe-delivery network through infrastructure upgrades and capacity building during the first year. The Year 1 HFAs conducted in July-August 2013 were used to assess infrastructure and capacity at the end of Year 1.

### Pregnancy outcome monitoring in facilities

Individual and aggregate retrospective pregnancy outcome data, including facility maternal deaths, were collected from delivering facilities by trained health staff and evaluation personnel. In Uganda, staff collected individual data on maternal, delivery—including obstetric surgeries—and newborn outcomes in facilities with comprehensive EmONC (CEmONC) [34]. They collected information on up to three maternal complications at the time of delivery, but only the most immediately life-threatening complication was used to analyze maternal morbidities and direct obstetric case fatality rates (CFR). Only aggregated reporting using specially designed extraction forms was employed in lower level facilities in Uganda and all facilities in Zambia, primarily from maternity registers. Surgical and admission/discharge registers were used in higher level facilities. Two districts in Zambia used broader aggregation of obstetric complications that precluded a separate examination of uterine rupture and abortion complications. In both countries, the number of maternal deaths in each facility was reconciled after crosschecking the reports of facility maternal deaths from communities.

### Rapid ascertainment process for institutional deaths (RAPID)

Detection of maternal deaths in 16 CEmONC facilities in Uganda was enhanced using the RAPID methodology [35]. This method includes review of all health facility records related to deaths among women of reproductive age (WRA) to reduce undercounting of maternal deaths and improve their notification, reporting and review. RAPID data collection using pilot-tested extraction forms was conducted retrospectively at two points in time (November 2012 and July 2013) in all 16 facilities by CDC epidemiologists in collaboration with facility-based teams.

### Reproductive age (12–49 years) mortality studies (RAMOS)

In Uganda, two retrospective RAMOS were conducted in the four SMGL districts to capture maternal deaths during the baseline (field work conducted in August–September 2012) and Year 1 (field work conducted in October–November 2013). The first step of each investigation, performed by village health teams (VHTs), consisted of identifying households in which a WRA had died. Trained VHTs compiled lists of deaths to WRA using their VHT registers. The second step consisted of a brief household investigation using a one-page screening tool to determine whether each WRA had been pregnant at the time of her death or in the 3 months preceding death. The third step consisted of interviewing the caretakers of women who died while pregnant or postpartum, using a special verbal autopsy (VA) questionnaire to explore causes and circumstances of maternal deaths [31, 36]. The VA instrument developed in Uganda is a new and complex verbal and social autopsy questionnaire that has been featured in the 2013 WHO technical guidance on maternal death surveillance and response [36]. Each death was certified and coded independently by two physicians, with a third if consensus was not reached. Only maternal deaths during pregnancy, delivery and 42 days postpartum that occurred during baseline and Year 1 periods were retained in the analyses.

In Zambia, the SMGL districts did not have an existing community-based data collection mechanism analogous to the village health teams in Uganda and RAMOS was not conducted.

### Population denominators

Calculation of population MMRs and selected process and outcome EmONC indicators (e.g. institutional delivery rate, C-section rate, and met need for obstetric care) requires external population data. District-wide censuses were conducted with SMGL support in Zambia in 2012 and in Uganda in 2013 to enumerate households, population, and women of reproductive age. Enumerations were projected back to estimate 2011 population using the inverse growth coefficient derived from the intercensal population growth provided by the national statistics bureaus. Live births were estimated by applying crude birth rates —directly derived from 2010 national census in Zambia and calculated by summing expected births among WRA in each age group multiplied by the rural age specific fertility rates from 2011 DHS in Uganda— to the baseline and Year 1 district populations.

The study protocol was reviewed by CDC’s Institutional Review Board and approved by CDC Human Research Protection Office of the Center for Global Health; it complied with Uganda and Zambia Ministries of Health procedures for protecting human rights in research. For conducting verbal autopsies, written informed consent among the caretakers of the deceased subjects was obtained after informing them about the purpose and public health importance of the research, selection procedures, voluntary participation and confidentiality. Interviews were scheduled no sooner than 6–8 weeks after the death occurred.

### Statistical analyses

The results shown here are based on 4-district data analyses performed for each country. They are based on the total population and total number of health facilities in the SMGL districts in each country. They are not a sample and are not representative of a larger population in the country.

Likewise the maternal mortality ratios presented are based on complete enumeration of all maternal deaths identified in facilities (Uganda and Zambia) and communities (Uganda) and thus not subject to sampling error, but may be affected by random variation and changes in case detection. In comparing the baseline and Year 1 periods, a z-statistic was used to calculate the *p*-value of the difference between the two MMRs, both in facilities and when comparing population MMRs [37]. Similarly, the changes in other core indicators, on the basis of complete counts of events during the two periods, were estimated using z-statistics for significance testing. The McNemar’s test, which is appropriate for dichotomous responses for matched pairs of data at different time points, was used to test for significance of changes between the baseline and Year 1 HFA indicators [38].

## Results

Access to care, infrastructure, and delivery care improved in SMGL districts following implementation of the SMGL-supported interventions (Table 5). Almost all delivering facilities (95%) in Uganda and Zambia provided delivery care 24 h/7 days per week by the end of Year 1. The proportion of facilities with uninterrupted electricity and water supplies increased significantly in both countries. In Zambia, the proportion of facilities with functional communication systems doubled and those performing community outreach increased by 35%. Availability of life-saving medications increased, with substantial reductions in facilities reporting stock-outs of magnesium sulfate and oxytocin in both countries. The practice of active management of the third stage of labor (AMTSL) increased significantly, becoming nearly universal (92–93%) in both countries. The proportion of facilities conducting maternal death reviews (MDR), mandated by official policies in both countries, rose by over 400% in Uganda, but did not change significantly in Zambia. The number of EmONC facilities providing basic or comprehensive interventions rose by 200 and 129% respectively in Uganda and by 100 and 25% in Zambia. The proportion of mid-level facilities performing 4–5 EmONC interventions increased by 57% in Uganda and by 54% in Zambia.

**Table 5:** Selected facility characteristics and interventions at Baseline and Year 1 SMGL

Characteristic/Intervention	Uganda (107 facilities)				Zambia (113 facilities)			
	Baseline^a^	Year 1^a^	% Change^b^	Sig. Level^c^	Baseline	Year 1	% Change^b^	Sig. Level^c^
Availability 24/7	80.4	95.3	19	***	68.1	94.7	40	***
Community outreach activities (Zambia only)	NA	NA	NA	NA	63.0	85.2	35	***
Electricity available	57.9	94.4	62	***	56.6	76.1	33	***
Water available	76.6	94.4	22	***	90.3	99.1	10	***
Functional communications available^d^	93.5	92.5	−1	NS	45.1	89.4	98	***
Transportation available5^f^	59.8	64.5	8	NS	54.9	61.1	11	NS
Sufficient number of beds	35.5	73.8	108	***	63.7	67.3	6	NS
Use of parenteral antibiotics in last 3 months	85.0	92.5	9	NS	78.8	75.2	−5	NS
Use of parenteral oxytocin in last 3 months	70.1	95.3	36	***	90.3	94.7	5	NS
Use of parenteral anticonvulsants in last 3 months	49.5	37.4	−24	NS	44.2	33.6	−24	NS
Perform newborn resuscitation in last 3 months	31.8	69.2	118	***	26.5	63.7	140	***
Perform manual removal of placenta in last 3 months	26.2	48.6	85	***	38.1	33.6	−12	NS
Remove retained products in last 3 months	18.7	50.2	168	***	16.8	38.1	127	***
Perform assisted vaginal delivery (AVD) in last 3 months	4.7	11.2	138	**	9.7	14.2	46	NS
Perform surgery (C-section) (HC IV or higher) in last 3 months	7.5	15.0	100	***	4.4	4.4	0	NS
Perform blood transfusion (HC IV or higher) in last 3 months	7.5	13.1	75	**	5.3	6.2	17	NS
Breech delivery performed in last 3 months	35.5	52.3	47	**	36.3	51.3	41	**
No stock out last 12 months: magnesium sulfate^f^	46.7	61.7	32	**	22.4	87.3	290	***
No stock out last 12 months: oxytocin^f^	56.1	82.2	47	***	78.2	97.5	25	***
HIV rapid test kits currently available^f,g^	71.0	82.2	16	NS	82.7	93.8	13	**
Active management of 3rd stage of labor (AMTSL)	75.7	92.5	22	***	70.8	91.2	29	***
Perform maternal death reviews	6.5	33.6	417	***	42.0	55.6	32	NS
Number of functioning CEmONC facilities	7	16	129	***	4	5	25	***
Number of functioning BEmONC facilities	3	9	200	***	3	6	100	***
Lower-level health facilities with partial BEmONC^h^	28	44	57	***	24	37	54	***

^a^Baseline period is June 2011–May 2012; Year 1 period is June 2012–May 2013

^b^Percent change calculations based on unrounded numbers

^c^ Asterisks indicate significance level of the difference between baseline and Year 1 outcomes for all facilities combined, using McNemar’s exact test, as follows: *** *p* < 0.01, ** *p* < 0.05, *NS* not significant

^d^Uganda: Facility owned landline, cell, two-way, or radio, or individual had cell phone. Zambia: Includes 2-way radio, landline, or cell phone with service

^e^Uganda: Available and functional motorized vehicle with fuel, funds for driver and maintenance generally available. Zambia: Includes motor vehicle, motorcycle, or bicycle

^f^Zambia: Kalomo facilities did not collect the information and were excluded from the analysis

^g^Uganda: Rapid HIV test was used in maternity ward in the last 3 months (does not indicated current availability)

^h^Percent of health centers (HC) that performed 4–5 basic emergency obstetric care interventions in the past 3 months

Note: Unless otherwise noted, the figures in the table are percentages of all facilities

Virtually all outcome indicators that SMGL sought to improve changed substantially between baseline and Year 1 (Table 6). Most noteworthy are the sharp increases in facility delivery rates in SMGL districts in both countries**.** In 1 year, the percentage of births that took place in health facilities rose by 62% (from 46 to 74%) in Uganda and by 35% (from 63 to 84%) in Zambia. The EmONC delivery rates increased by 28 and 17%, respectively. Cesarean section rates among all births in the SMGL districts increased by 23% in Uganda and 15% in Zambia.

**Table 6:** Pregnancy and maternal health outcomes in facilities at baseline and during Year 1 SMGL

Pregnancy and Maternal Health Outcomes	Uganda			
	Baseline	Year 1	% Change	Significance^a^
Number of live births – All facilities	33,492	56,571	69	***
Institutional delivery rate - All facilities (%)	45.5	73.8	62	***
Institutional delivery rate - EmONC facilities (%)	28	36	28	***
Number of obstetric complications treated^b^	5249	7696	47	***
C-section rate as a proportion of all births (%)	5.3	6.5	23	***
Met need for emergency obstetric care -All facilities (%)	46	66	42	***
Met need for emergency obstetric care -EmONC facilities (%)	39	49	25	***
Direct Obstetric Case Fatality Rate (%)	2.6	2.0	−25	***
Direct Maternal Mortality Ratio (MMR)	416	269	−35	***
Facility MMR, overall	534	345	−35	***
Obstetric hemorrhage MMR^c^	131	94	−29	***
Puerperal infection/Sepsis MMR	75	32	−57	**
Obstructed labor MMR^d^	72	30	−58	***
Abortion-related MMR^e^	63	35	−44	NS
Pre-eclampsia/Eclampsia MMR	45	46	3	NS
Other Direct Obstetric Causes MMR^f^	30	32	7	NS
Indirect Obstetric Causes MMR^g^	119	76	−36	NS
	Zambia			
Number of live births –All facilities	21,914	30,619	40	***
Institutional delivery rate (%)	62.6	84.3	35	***
Institutional delivery rate-EmONC facilities (%)	26	30	17	***
Number of obstetric complications treated^b^	1833	2462	34	***
C-section rate as a proportion of all births (%)	2.7	3.1	15	***
Met need for emergency obstetric care -All facilities (%)	34	45	31	***
Met need for emergency obstetric care -EmONC facilities (%)	26	32	23	***
Direct Obstetric Case Fatality Rate (%)	3.1	2.0	−34	**
Direct Maternal Mortality Ratio (MMR)	260	166	−36	**
Facility MMR, overall	310	202	−35	**
Obstetric hemorrhage MMR^c^	110	72	−34	NS
Obstructed labor MMR^d^	59	13	−78	**
Other Direct Obstetric Causes MMR^f^	91	82	−11	NS
Indirect Obstetric Causes MMR^g^	50	36	−28	NS

^a^Asterisks indicate significance level of the difference between baseline and Year 1 outcomes for all facilities combined, using a z-statistic to calculate the *p*-value of the difference, as follows: *** *p* < 0.01, ** *p* < 0.05, *NS* not significant

^b^Excludes first-trimester complications (e.g. abortion-related complications and ectopic pregnancy)

^c^Includes antepartum, intrapartum and postpartum hemorrhage

^d^Obstructed and prolonged labor including rupture of the uterus

^e^Includes both induced and spontaneous abortions

^f^Includes embolism, anesthetic-related deaths, and ectopic pregnancy

^g^Includes HIV-, TB- and malaria-related maternal deaths, and those due to other medical conditions

The number of complicated deliveries treated in facilities also increased in both countries. The overall proportion of expected obstetric complications treated (based on an assumed incidence of complications during pregnancy and childbirth of 15%) increased by 42% in Uganda and by 31% in Zambia. The proportion of expected complications treated in EmONC facilities grew by 25% in Uganda and 23% in Zambia. The direct obstetric CFR declined by 25% in Uganda, and by 34% in Zambia.

Facility-based MMRs fell by 35% in the SMGL districts in each country—from 534 to 345 deaths per 100,000 live births in Uganda and from 310 to 202 in Zambia. In Uganda, the facility MMR declined significantly between baseline and Year 1 for three major direct obstetric causes: obstetric hemorrhage (29%); obstructed labor (58%); and postpartum sepsis (57%). In Zambia, only maternal mortality due to obstructed labor fell significantly (78%).

Population-based maternal mortality in Uganda SMGL districts are based on information collected through verbal autopsies (Table 7). Only 6 suspected maternal deaths identified in the baseline RAMOS and 5 deaths in the Year 1 RAMOS were not followed by an interview due to household dissolution or relocation. There were no refusals to participate in the RAMOS studies. The number of maternal deaths dropped from 342 to 247 and MMR declined significantly in Year 1—from 452 deaths per 100,000 live births to 316 deaths, a reduction of 30%. Significant declines in cause-specific mortality were observed for obstetric hemorrhage (43%), obstructed labor (54%), and sepsis (49%). Mortality fell significantly during the intrapartum and up to 24 h post-partum period (27%) and between 1 and 42 days after delivery (40%). No significant change occurred in mortality before the onset of labor.

**Table 7:** Changes in district-wide Maternal Mortality Ratio (per 100,000 Live Births) by cause, timing of death, and the Three Delays: Uganda SMGL Districts

	Maternal Mortality Ratio (MMR)			
	Baseline	Year 1	% Change	Significance^a^
Total^b^	452	316	−30	***
Causes of Death				
Obstetric Hemorrhage	128	73	−43	***
Pre-eclampsia/Eclampsia	58	45	−22	NS
Obstructed Labor (Including Uterine Rupture)	71	33	−53	***
Puerperal infection/Sepsis	33	17	−48	**
Abortion-related	42	36	−14	NS
Other Direct Obstetric Causes	49	31	−37	NS
Indirect Causes	70	82	17	NS
Timing of Death				
Antepartum	62	60	−3	NS
Intrapartum and Immediate Postpartum (up to 24 h)	168	121	−28	**
> 24 h-42 days Postpartum	222	134	−40	***
The Three Delays				
Delays in seeking care	124	66	−47	***
Delays in reaching care	40	16	−60	***
Delays in receiving care (one hour or more)	92	54	−41	***

^a^Asterisks indicate significance level of the difference between baseline and Year 1 MMRs, using a z-statistic to calculate the *p*-value of the difference, as follows: *** *p* < 0.01, ** *p* < 0.05, *NS* not significant

^b^Baseline MMR = 342 maternal deaths/75,675 live births*100,000; Year 1 MMR = 247 maternal deaths/78,261 live births *100,000

Substantial reductions were reported in the MMR in Uganda for all three major delays that can cause maternal death: mortality from delays in deciding to access appropriate care decreased by 46%; from delays associated with reaching care fell by 61%; and from delays in receiving quality care after reaching a facility declined by 43%.

## Discussion

A comprehensive health-system strengthening approach with improvements in access to, availability of, and quality of maternity care in the SMGL districts in the first year of the initiative was associated with a 30% decline in population-based maternal mortality in Uganda and a 35% decline in MMR in Ugandan and Zambian health facilities. To our knowledge reductions in maternal mortality of this magnitude in such a short time are unprecedented in SSA. Improvements were also reported in nearly all indicators of maternal health.

Substantial increases in institutional delivery rates were an important contributing factor and are associated with encouraging communities to promote facility delivery (through community health workers and local leaders), improving transportation, preparing facilities for increased demand, and improving the quality of services [29, 30]. The rate increased more in Uganda than in Zambia (62 vs. 35%), suggesting the contribution of subsidized transport vouchers (introduced only in Uganda). However the Year 1 facility delivery rates in Zambia were higher (84%) than in Uganda (63%).

About 15% of women are estimated by WHO to develop pregnancy complications and require timely access to emergency obstetric care [14]. The number of fully functional EmONC facilities providing such care and the EmONC delivery rate rose significantly in both countries, an indication of increased capacity to better respond to obstetric emergencies. As more women with direct obstetric complications accessed EmONC, the met need for emergency obstetric care increased. The increase in met need documented here (42% in Uganda and 31% in Zambia) is a conservative estimate because it did not take into account first trimester complications treated in facilities (including post-abortion complications, whose severity could often not be determined); nor did it take into account treatment of complications developed several days after delivery (e.g. postpartum sepsis) that were admitted directly to the postpartum ward. Care for these complications increased during Year 1 due to better access and referral pathways [29].

The EmONC improvements enhanced capacity for surgical obstetric care. Each country reported a significant increase in the population C-section rate. However only Uganda’s Year 1 rate of 6.5% was within the 5–15% range recommended by WHO. It should be noted that although the C-section rate increased, information is not available to examine how appropriate the decision for this route of delivery was and if any alternative management attempts were made (e.g. expected management, AVD, manual rotation of the occiput). Further special studies would be necessary to evaluate the quality of C-section deliveries in SMGL districts.

Availability of emergency obstetric care within 2 h of the onset of a complication, improved management and supply chains, routine use of AMTSL, and rising C-section rates are indications of improved quality of obstetric care [14]. These improvements led to declines in the CFRs for direct obstetric complications treated in facilities, though they still exceeded the WHO recommended level of <1%. The CFRs reported here are conservative estimates because first trimester complications were not included in the CFR denominators while deaths that occurred following these complications were captured in the numerators.

Obstetric hemorrhage declined significantly in Uganda, in facilities and the population as a whole, likely associated with the increase in use of AMTSL, manual removal of placenta, removal of retained products and availability of blood transfusions and obstetric surgery. Nevertheless, it remained the leading cause of maternal death in both countries. In Uganda, obstructed labor (including uterine rupture) and sepsis (often due to prolonged labor) showed the sharpest declines in both facilities and in the population, likely due to the increased availability of C-sections. All other causes of death did not change significantly. The causes of death identified in this analysis are similar to WHO cause-of-death estimates, but differ from the Global Burden of Disease (GBD) estimates, which identify induced abortion as the greatest single contributor to maternal mortality in Eastern SSA [5, 11, 39].

The 30% decline in population-based maternal mortality in Uganda provides the most credible evidence of the success of the SMGL initiative in Year 1. Declines in mortality were significant during delivery and immediately postpartum (28%), when SMGL interventions would be expected to have their greatest impact. The largest decline occurred in mortality from 25 h to 42 days postpartum (40%) and was likely rooted in a better management of women during labor and delivery, which prevents complications that extend longer post-partum such as sepsis. A shift in the place of death (from 48% of maternal deaths occurring in facilities at baseline to 63% in Year 1) [31] is evidence that more women with severe complications are reaching health facilities, some accessing care many hours after complicated home deliveries and possibly not getting there soon enough while others receiving lifesaving postpartum care. The timing of death in relationship to labor and delivery is similar to the 2013 GBD reports, which estimate that 26% of maternal deaths worldwide occur intrapartum and immediately postpartum and 49% during 25 h to 42 days after delivery.

Although facility-based MMR data can be used to improve health planning and quality of care, this information should not be viewed as representative of the districts as a whole. Facility-based MMRs may be higher or lower than those reported in the general population depending on the mix of patients (with more or fewer severe complications), the timeliness of medical care, the quality of the care, the proportion of deliveries at facilities, and the length of stay post-delivery. Facility-based MMRs are also subject to selection bias because they include only women who accessed obstetric care services.

Declines in the CFR contributed to the reductions in MMRs. CFR is largely used as a proxy outcome indicator to signal changes in quality of care in EmONC services [14]. This decline shows that life-saving emergency obstetric care provided for complicated deliveries was effective and the quality of care improved despite a large increase in the volume of patients attended and the number of complicated deliveries.

The examination of MMR using a “Three-Delay” framework can help identify the relative contribution of health care decision-making, accessing care, and quality of childbirth services to the elimination of preventable maternal deaths. SMGL interventions were associated with substantial reductions in MMRs for all three of the major delays, suggesting that comprehensive community and facility approaches are needed for significant maternal mortality reductions to occur.

In settings with limited registration of births and deaths and incipient health information systems, monitoring of maternal mortality is largely done through model-based estimates [11, 39]. Country-owned, real-time, district level direct measurements of maternal mortality in Uganda and Zambia were made possible, for the first time, through the SMGL initiative. This assessment employed multiple methods to detect changes in maternal mortality in communities and facilities. It provided information about the processes and outputs that mediated maternal death declines and triggered countries’ decisions to support scale-up of the SMGL approach.

This evaluation compares intervention districts before and after SMGL implementation, without a control in non-intervention districts. SMGL was rapidly launched as a full-coverage initiative, so an appropriate control group within the pilot districts could not have been established. Each country implemented district-level interventions with similar scope, timelines and intensity and adopted identical monitoring and evaluation indicators. However, interventions were prioritized and implemented differently, reflecting existing country contexts and needs. For example, Zambia did not have a community-based system to collect health data in place before the SMGL initiative began. It set up a new system of community key informants (Safe Motherhood Action Groups or SMAGs), tasked to track each pregnant woman until the end of the 42-day postpartum period, but logistic challenges limited the coverage. Similarly, data availability and quality in health facilities varied between and within each country. Although the absence of control districts introduced inherent limitations in our ability to attribute positive health outcomes to the SMGL interventions, significant improvements did occur in most outcomes in SMGL districts in both countries, despite differences in the measurement approaches.

The comparison highlights accomplishments, particularly maternal mortality declines, likely associated with the introduction of the SMGL model. The recently estimated overall decline in Uganda has been about 3% per year [4], making it extremely unlikely that the 30% reduction in Uganda’s SMGL districts was unrelated to the SMGL interventions.

The observed decline in MMRs in this analysis is conservative. Since SMGL’s investments started before the official launch in June 2012, the baseline period actually includes several months of building up to the full-fledged model, meaning that the documented decline in MMR is in all likelihood an underestimate. Furthermore, the completeness of mortality data depends on the accuracy of causes of death determination. Improvements in data accuracy mediated by SMGL rendered the baseline results not entirely comparable with the Year 1 results. Several factors, including lack of information about the antenatal period, lack of 24/7 laboratory diagnostics, limited experience with classification and coding of deaths, and low utilization of MDRs prior to Year 1 may have contributed to a greater extent to underreporting of maternal deaths at baseline than in Year 1, resulting in a smaller observed decline in MMR. Retrospective data collection on pregnancy and mortality outcomes in facilities and in communities does not allow for a detailed evaluation of the quality of care received. In the absence of patient records, case management details on complicated deliveries, discharge notes, or direct observation of practices, information from registers lack details about prenatal period, the time between admission and delivery, if complications were present at admission, for how long, and how severe they were, whether a complete and timely assessment of the status at admission was performed, and what was the quality of monitoring and care during labor and delivery. Smaller scale quality of care studies in a subset of SMGL-supported facilities are proposed to help document quality of care and remaining gaps.

## Conclusions

Maternal mortality fell significantly in 1 year in eight pilot districts in Uganda and Zambia following the introduction of the SMGL model. This decline is likely due to parallel improvements of the supply- and demand-side for obstetric and HIV services coupled with improved quality of care at facilities and improved coordination and health management throughout the districts. Although implementation and emphasis of SMGL interventions were not identical in each district, maternal health outcomes in facilities improved in both countries.

In Uganda, the 30% population-based decline in maternal mortality was accomplished through a comprehensive district system strengthening approach that led to reductions in the “Three Delays.” Maternal mortality reductions in these countries of such a magnitude in 1 year show that it is possible to greatly accelerate progress in saving mothers’ lives. The lessons learned from SMGL can inform policymakers and program managers in other low and middle income settings where similar approaches could be utilized to rapidly reduce maternal mortality.

## Abbreviations

AMTSL: Active management of the third stage of labor
AVD: Assisted vaginal delivery
BEmONC: Basic emergency obstetric and newborn care
CDC: Centers for Disease Control and Prevention
CEmONC: Comprehensive emergency obstetric and newborn care
CFR: Case fatality rate (for direct obstetric complications receiving care)
C-section: Cesarean section
DHS: Demographic Health Survey
EmONC: Emergency obstetric and newborn care
GBD: Global burden of disease
HFA: Health facility assessment
HIV: Human immunodeficiency virus
ICD: International Statistical Classification of Diseases and Related Health Problems
M&E: Monitoring and evaluation
MDG: Millennium development goal
MDR: Maternal death review
MMR: Maternal mortality ratio
NMR: Neonatal mortality rate (in health facilities, pre-discharge)
PMR: Perinatal mortality rate (in health facilities, pre-discharge)
RAMOS: Reproductive age mortality study
RAPID: Rapid ascertainment process for institutional deaths
SMAG: Safe motherhood action group
SMGL: Saving mothers, giving life
SSA: sub-Saharan Africa
VA: Verbal autopsy
VHT: Village health team
WHO: World Health Organization
WRA: Women of reproductive age (15–49 years)
